# A Comparative Study of High-Resolution Chemical-Shift-Eliminated Magnetic Resonance Imaging of Finger Specimens with Microcomputed Tomography

**DOI:** 10.25259/JCIS-20-2019

**Published:** 2019-05-24

**Authors:** Wingchi Edmund Kwok, Zhigang You, Johnny Monu, Hua He

**Affiliations:** 1 Department of Imaging Sciences, University of Rochester, Rochester, New York, USA; 2 Department of Epidemiology, Tulane University School of Public Health and Tropic Medicine, New Orleans, Louisiana, United States.

**Keywords:** Bone imaging, Chemical-shift artifacts, Finger joint imaging, High-resolution magnetic resonance imaging, Magnetic resonance imaging

## Abstract

**Objective:**

High-resolution images of finger joints with chemical-shift elimination can be obtained using an interleaved water-fat (IWF) sequence. This study assessed IWF imaging of finger joints in the delineation of bone structures by comparing images of cadaver fingers with those of microcomputed tomography (CT) that served as a standard reference.

**Materials and Methods:**

IWF images with spatial resolution of 176 µ × 176 µ × 300 µ were obtained from the distal and proximal interphalangeal joints of two cadaver finger specimens using a custom-built radiofrequency receive coil at 1.5T. Regular three-dimensional gradient-echo (GRE) images were also acquired with similar parameters and compared with the IWF images to evaluate the effects of chemical shift. Micro-CT scans were obtained and served as the standard reference. The image data were reviewed by two experienced musculoskeletal radiologists in consensus. The delineation of normal joint structures and abnormalities in the finger specimens as revealed by the magnetic resonance imaging (MRI) and micro-CT images were compared. The IWF and regular GRE images were assigned scores 0–3 for the depiction of apparent marginal bone defects, with zero being the same in appearance to the micro-CT image and three as having minimal resemblance to it. Statistical analysis of the scoring results was conducted to compare the two MRI techniques.

**Results:**

The high-resolution IWF images provided accurate delineation of bone and calcified structures as seen in micro-CT. The thickness of subchondral bone was depicted similarly on the IWF water + fat and the micro-CT images but not on the regular GRE images. The regular GRE sequence showed false marginal bone defects not observed with IWF and micro-CT. In addition, the IWF water-only images facilitated the identification of bone cyst by revealing its water content.

**Conclusion:**

High-resolution IWF imaging should be useful for the early diagnosis and treatment assessment of arthritis and should also benefit basic research in the pathophysiology of the disease.

## INTRODUCTION

Magnetic resonance imaging (MRI) is more sensitive than plain radiographs in detecting arthritis that will benefit from early aggressive treatment.^[1-3]^ Finger joints are of particular interest for arthritis examinations since they are commonly involved^[4,5]^ and are often the first joints affected. However, MRI of finger joints is difficult due to their small size. Partial volume effects hinder the visualization of lesions^[6]^ and the bone.^[7]^ Accurate assessment of the bone is important as para-articular erosion portends irreversible joint damage.^[8]^ Patients with bone damage at an early stage of arthritis need to be identified and monitored closely.^[9]^ To provide the optimal signal and image resolution for MRI of finger joints, dedicated radiofrequency (RF) coils were developed.^[10]^

Another obstacle to finger MRI is chemical-shift artifacts that hinder the evaluation of bone and other structures^[11,12]^ and at times resemble erosive changes.^[7,13]^ Studies found mismatches in erosion detection between MRI and computed tomography (CT).^[14,15]^ In addition, chemical-shift artifacts alter the appearance of subchondral bone^[11,13,16]^ and may affect the early detection of osteoarthritis.^[17]^ To eliminate chemical-shift artifacts, an interleaved water-fat (IWF) sequence had been developed^[18]^ and applied to high-resolution finger imaging.^[13]^ It acquires both water-only (fat-suppressed) and fat-only images and eliminates chemical-shift artifacts in the combined water + fat (nonfat-suppressed) images. The objective of this study was to evaluate high-resolution IWF imaging of finger joints in the depiction of bone structures by acquiring images of cadaver fingers and compared with micro-CT.

## MATERIALS AND METHODS

Two cadaver finger specimens obtained from the National Disease Research Interchange (NDRI, Philadelphia, PA, USA) were used. According to the information provided by NDRI, they were harvested from two donors – a normal finger from a 68-year-old male and an arthritic finger from a 71-year-old male with unspecified arthritis. These fingers were among the second-to-fourth digits of the hand. They were preserved in a freezer and were thawed at room temperature before the use. The Institutional Biosafety Committee of our institution approved this study.

MR imaging was conducted on a General Electric (Milwaukee, WI) 1.5T scanner with maximum gradient amplitude of 40 mT/m and maximum slew rate of 150 T/m/s. An RF receive coil specially developed for finger joints was used to obtain high-resolution images. The coil consisted of a copper tape loop 2.9 cm in diameter and 2.0 cm in width and had a similar design as the one described in Reference 10. For scanning, the coil was placed with its axis perpendicular to the main magnetic field. The finger specimen was inserted through the coil with the joint being imaged centered in the coil. The distal interphalangeal (DIP) and proximal interphalangeal (PIP) joints were imaged separately.

**Figure 1:**
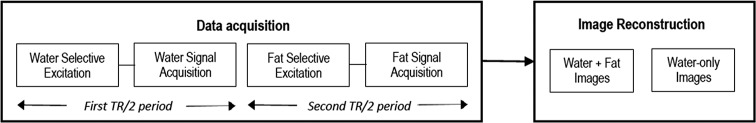
Schematic diagram of the interleaved water-fat sequence used in this study. By acquiring water-only and fat-only signals in an interleaved fashion within each repetition time period, the interleaved water-fat sequence provides both water + fat (nonfat-suppressed) and water-only (fat-suppressed) images in a single-scan time.

An IWF sequence was modified from the regular three-dimensional gradient-echo (3D GRE) sequence through pulse sequence programming.^[13,18]^ It excited and acquired water-only and fat-only signals in an interleaved manner during each repetition time (TR) period [Figure 1]. Sagittal and coronal images of the finger joints were acquired with TR 52 ms, echo time 12 ms, field of view (FOV) 4.5 cm, acquisition matrix 256 × 256, in-plane resolution 176 µm × 176 µm, slice thickness 300 µm, flip angle 20°, default receive bandwidth ± 15.63 kHz (122 Hz/pixel), and scan time 14.2 min. Frequency-encoding direction was along the axis of the finger specimen, and the polarity of the frequency-encoding gradient was selected such that the proximal side of the finger experienced a higher magnetic field. Regular 3D GRE images with and without fat suppression were also acquired using similar parameters for comparison. To enable the thin-slice acquisition, the RF pulse width of the regular GRE sequence was increased from 3.2 ms to 6.4 ms through pulse sequence programming. The IWF data were reconstructed into water + fat (nonfat-suppressed) and water-only (fat-suppressed) images using a custom MATLAB (MathWorks, Natick, MA, USA) program on a remote computer. The in-plane matrix size was increased to 1024 × 1024 using bicubic interpolation. The regular GRE images were also reconstructed on MATLAB with similar settings to allow fair comparison with the IWF images.

For the micro-CT study, a Viva CT40 system (Scanco Medical AG, Basserdorf, Switzerland) was used. Images were obtained in the transverse plane with FOV 19.2 mm × 18.3 mm at a resolution of 30 µm × 30 µm ×30 µm per voxel using a voltage 55 kVp, current 142 µA, and an integration time of 200 ms.

Osirix software (http://www.osirix-viewer.com/) was used to process the high-resolution MRI and the micro-CT data for evaluation. To facilitate comparison of the two modalities, the micro-CT image data were reformatted to match the MR images in slice position and orientation. Image evaluation was performed by two experienced musculoskeletal radiologists in consensus. Since the purpose of the study was to assess the depiction of bone structures, only the water + fat or nonfat-suppressed MR images were evaluated. Each set of the IWF water + fat and regular GRE nonfat-suppressed MRI data was compared to the corresponding micro-CT image on a computer monitor, with micro-CT on top and the two MR techniques at the bottom with randomized left-right position. Five MRI slices in the coronal plane and another five in the sagittal plane from each finger joint were selected for the comparison. For each plane, the five selected slices comprised one slice in the center of the joint plus the fifth and tenth slices on either side of it. The distance between adjacent selected slices was 1.5 mm.

Since the DIP of one of the fingers spanned only over three selected slices in the coronal plane, the total number of image sets in the comparison was 38. The MRI data were assigned scores between zero and three for the depiction of apparent marginal bone defects, with zero being the same in appearance to the micro-CT image and three as having minimal resemblance to it. The proximal and distal sides of each finger joint were scored separately. Statistical analysis of the scoring results was conducted to compare the two MRI techniques using the Statistical Analysis Software (SAS Institute, Cary, North Carolina, USA). Specifically, the proportional-odds cumulative logistic regression models were employed to model the ordinal outcome with the probability of having a lower score (better resemblance to the micro-CT). The proportional-odds assumption was also checked. For each side of the joint (distal or proximal), comparisons were made between (1) the two MRI techniques in general and (2) between the two MRI techniques at either the sagittal or coronal plane to evaluate the dependency of the imaging performance on slice orientation. The odds ratio (OR) of having the lower score, *P* value, and 95% confidence interval (CI) of OR were provided for each set of the comparison.

## RESULTS

**Figure 2:**
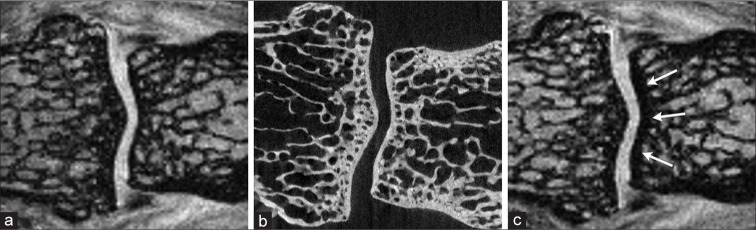
The subchondral bone thickness of this proximal interphalangeal joint was depicted similarly on the coronal (a) interleaved water-fat water + fat and (b) microcomputed tomography images. However, it appeared to be much thicker on the proximal side of the joint (arrows) on the (c) regular nonfat-suppressed gradient-echo image.

**Figure 3:**
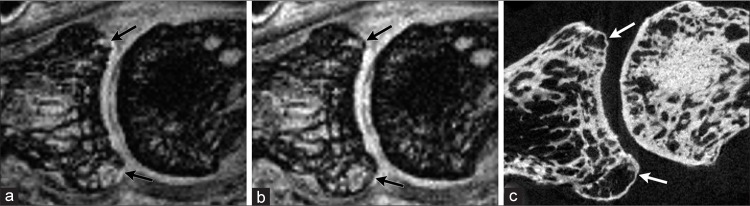
There were apparent disruptions of the marginal bone (arrows) seen on the sagittal (a) regular gradient-echo nonfat-suppressed image of this proximal interphalangeal joint but not on the corresponding (b) interleaved water-fat water + fat image. The (c) microcomputed tomography image confirmed the marginal bone was intact.

**Figure 4:**
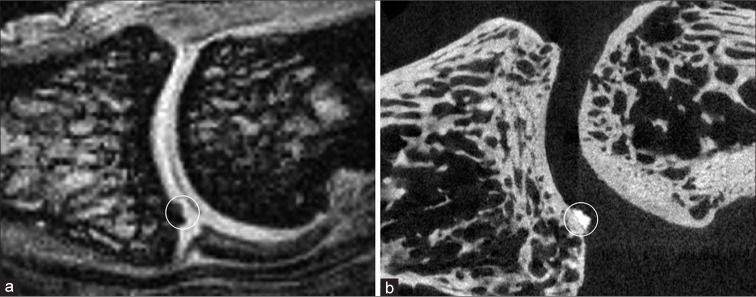
In this proximal interphalangeal joint, calcification inside the cartilage (circle) was observed on the sagittal (a) interleaved water-fat water + fat image as on the (b) microcomputed tomography image.

**Figure 5:**
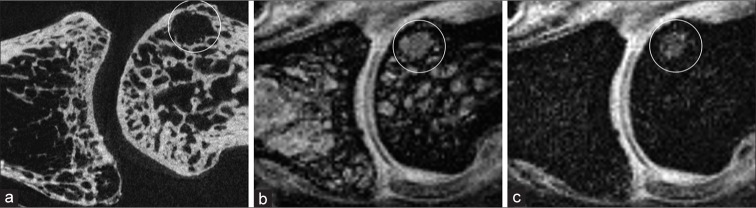
A subchondral defect (circle) was seen in this proximal interphalangeal joint on the sagittal (a) microcomputed tomography and (b) interleaved water-fat water + fat images. The (c) interleaved water-fat water-only image revealed its water content and identified it as a cyst.

As mentioned in the Method section, four finger joints (2 DIP and 2 PIP) were evaluated in this study. The high-resolution MRI and micro-CT finger images were able to be matched closely to each other, allowing precise comparison of the two modalities [Figures 2-5]. The IWF technique revealed detailed bone structures and abnormalities similar to those observed in micro-CT.

The thickness of subchondral bone was depicted similarly on the IWF water + fat and the micro-CT images. On the regular GRE nonfat-suppressed images, subchondral bone appeared thicker on the proximal side of the joint in the regular GRE images [Figure 2]. This is a result of chemical-shift phenomenon shifting cartilage and nearby joint tissues with water signal away from the bone marrow with fat signal on that side of the joint.

For the comparison of apparent marginal bone defects [Tables 1 and 2], the IWF sequence performed much better than the regular GRE sequence on the distal side of the joint (*P* = 0.0014; OR = 5.285, 95% CI = [1.90, 14.691]), and this was also the case when each imaging plane (sagittal or coronal) was considered separately (*P* = 0.0012; OR = 6.017; 95% CI = [2.029, 17.847]). The regular GRE sequence showed false marginal bone defects not observed with IWF and micro-CT [Figure 3], which were attributed to chemical-shift artifacts that brought neighboring joint tissues with water signal closer to the bone marrow (fat signal) on that side of the joint creating pseudo disruptions in the subchondral bone. On the other hand, IWF and regular GRE performed equally well in the depiction of marginal bone defects on the proximal side of the joint, being in complete agreement with micro-CT. This result is expected as pseudo thickening of the subchondral bone on the proximal side of the joint due to chemical-shift artifacts should not lead to the appearance of false defects at bone margins.

**Table 1:** Comparison of coronal interleaved water-fat and regular gradient-echo sequences in the depiction of apparent marginal bone defects.

Scores	Number of finger joint structures in each sequence							
	IWF (water + fat)				Regular GRE (nonfat-suppressed)			
	Distal		Proximal		Distal		Proximal	
	DIP	PIP	DIP	PIP	DIP	PIP	DIP	PIP
0 (best)	8	10	8	10	3	8	8	10
1	0	0	0	0	2	2	0	0
2	0	0	0	0	3	0	0	0
3	0	0	0	0	0	0	0	0

GRE: Gradient echo, DIP: Distal interphalangeal, PIP: Proximal interphalangeal, IEF: Interleaved water-fat

**Table 2:** Comparison of sagittal interleaved water-fat and regular gradient-echo sequences in the depiction of apparent marginal bone defects.

Scores	Number of finger joint structures in each sequence							
	IWF (water + fat)				Regular GRE (nonfat-suppressed)			
	Distal		Proximal		Distal		Proximal	
	DIP	PIP	DIP	PIP	DIP	PIP	DIP	PIP
0 (best)	5	8	10	10	4	2	10	10
1	4	1	0	0	3	7	0	0
2	1	1	0	0	3	1	0	0
3	0	0	0	0	0	0	0	0

GRE: Gradient echo, DIP: Distal interphalangeal, PIP: Proximal interphalangeal, IWF: Interleaved water-fat

The ability of IWF to reveal other abnormalities in finger joints was also demonstrated in this study. Articular cartilage calcification was observed on IWF water + fat images as seen with micro-CT [Figure 4a-c]. A bone cyst was better defined on the IWF than on micro-CT [Figure 5a-c]. Although the cyst was seen on both the IWF water + fat and the micro-CT images, it could not be clearly distinguished from the neighboring trabecular structure on the micro-CT image. In contrast, IWF provided additional water-only images that revealed its water content and confirmed its being a cyst instead of fatty marrow inside the trabecular structure [Figure 5c].

## DISCUSSION

This study demonstrated the ability of high-resolution IWF technique to accurately delineate bone structures in finger joints by comparing IWF and regular GRE images of cadaver finger specimens with micro-CT that served as the reference standard.

Our results indicate that, with the removal of chemical-shift artifacts, high-resolution IWF imaging can improve the depiction of bone and calcified structures in finger joints and avoid the false appearance of marginal bone defects as observed in the regular GRE images. In addition, IWF also provides water-only images that enable water content inside bone structures such as bone cyst to be clearly identified and distinguishes them from the fatty bone marrow in trabecular structures without the need of any signal intensity/density measurements as in CT.

Although utilizing higher receive bandwidth can reduce chemical-shift artifacts, this approach is not desirable for high-resolution imaging due to the associated decrease in signal-to-noise ratio (SNR) and the increase in gradient amplitude that limits spatial resolution. On the other hand, while pulse sequence techniques such as IDEAL^[19]^ can eliminate chemical-shift artifacts, they require multiple acquisitions that increase scan time.

In this study, micro-CT was used as the reference standard instead of histology since our focus was in bone and calcified structures, and CT-based techniques are often considered the gold standard for bone evaluation.^[14]^ Besides, micro-CT offers several advantages over histology. It can be used to visualize the whole finger joint, and its 3D data acquired with resolution in tens of microns can be reformatted to match closely with the corresponding MRI in any plane. In contrast, histology provides only a limited number of cross sections in a given plane, and the FOV may cover only a small portion of the joint.^[5]^ Furthermore, micro-CT is noninvasive and does not damage or change the joint structures as in histology.

While CT can provide clear depiction of bone structures in finger joints, it is less desirable for clinical use due to its ionizing radiation and its relatively low image contrast in soft and connective tissues. On the other hand, although ultrasound has been used for imaging finger joints, it is less sensitive in detecting bone erosions compared to MRI. In a study comparing ultrasound with CT of finger joints, small erosive lesions of <2 mm were found to cause false-negative results in ultrasound, whereas osteophytes mimic erosive lesions and led to false-positive results.^[20]^

A potential limitation of the high-resolution IWF imaging in this study is the relatively long scan time of 14.2 min. It results from the use of the 3D matrix size of 256 × 256 × 64 and longer TR to achieve the high image resolution. For the regular nonfat-suppressed and fat-suppressed scans, the minimum TR values are 23 ms and 40 ms, respectively. For the IWF scan, the minimum TR is further lengthened to 52 ms to accommodate the separate water and fat acquisitions in each TR period (this TR of 52 ms was also used in the regular GRE scans in this study to facilitate image comparison with the IWF scans). Despite the longer associated scan time, the longer TR is actually advantageous in that it provides higher signal to support the high-resolution acquisition. We found in a separate test that by increasing TR from the minimum value of 23 ms to 52 ms in the regular nonfat-suppressed GRE scan, SNR in the muscle was increased by about 50%. Furthermore, the IWF sequence provides both water + fat and water-only images in a single scan, thus saving the time to acquire both the conventional nonfat-suppressed and fat-suppressed scans.^[18]^ The minimum scan time for the regular nonfat-suppressed and fat-suppressed scans is 6.4 min and 11.0 min using the minimum TR of 23 ms and 40 ms, respectively, resulting in a combined minimum scan time of 17.4 min that is longer than the IWF scan of 14.2 min. The scan time for IWF may be reduced by using partial echo acquisition, though the effect to SNR and image quality will need to be assessed.

Another limitation of the technique used in this study is that each finger joint was imaged separately due to the custom single-loop RF coil used, and this restricts its potential clinical applications. Recently, a new flexible hand coil that provides high signal and full coverage for all fingers in a hand has been reported.^[21]^ Dedicated hand/fingers phased array coils such as this may enable high-resolution IWF imaging of multiple fingers.

## CONCLUSION

This study has characterized and verified the high-resolution IWF imaging findings in the bony and calcified structures of finger joints. This technique is able to reveal detailed structural information of finger joints that may not be seen with conventional MRI, and its elimination of chemical-shift artifacts provides accurate depiction of bone structures and avoids the false appearance of erosions. Potential applications of the technique include early diagnosis, monitoring of disease progression, and evaluation of the effects of early treatment of arthritis. This technique should also be useful for basic research in the pathophysiology of arthritis, such as early disease mechanisms in bone and cartilage, which may lead to improved treatment of the disease.
